# Suicide and deliberate self-harm in Pakistan: a scoping review

**DOI:** 10.1186/s12888-017-1586-6

**Published:** 2018-02-12

**Authors:** Sualeha S. Shekhani, Shagufta Perveen, Dur-e-Sameen Hashmi, Khawaja Akbar, Sara Bachani, Murad M. Khan

**Affiliations:** 1 Department of Psychiatry, Aga Khan University, Karachi, Pakistan; 2 Department of Community Health Sciences, Aga Khan University, Karachi, Pakistan; 3 Aga Khan University, Karachi, Pakistan; 4 Drexel University College of Medicine/Hahnemann University Hospital, Philadelphia, PA USA

**Keywords:** Pakistan, Muslim, Suicidal behavior, Public health

## Abstract

**Background:**

Suicide is a major global public health problem with more than 800,000 incidents worldwide annually. Seventy-five percent of the global suicides occur in low and middle-income countries (LMICs). Pakistan is a LMIC where information on suicidal behavior is limited. The aim of the review is to map available literature on determinants, risk factors and other variables of suicidal behavior in Pakistan.

**Method:**

This study was based on Arksey and O’Malley’s methodological framework of scoping review, combining peer reviewed publications with grey literature. Ten databases including Applied Social Sciences Index and Abstracts (ASSIA), Cochrane Trials Register (CRG), Cumulative Index to Nursing and Allied Health (CINAHL), National Library of Medicine Gateway (NLMG), ExcerptaMedica (EMBASE), National Library of Medicine’s MEDLINE (PUBMED), PSYCHINFO, Social Science Citation Index and Science Citation Index (SCI) and Pakmedinet.com were searched from the beginning of their time frames until December 2016 using a combination of key terms. The inclusion criteria included studies of various study designs covering different aspects of suicidal behavior in English language.

**Results:**

Six hundred and twenty three articles were initially retrieved from all ten databases. Two independent reviewers screened the titles and abstracts for relevance. One hundred and eighteen articles were read in full, out of which 11 were excluded because they did not fit the eligibility criteria. One hundred and ten articles, including two student theses and one report, were included in the final review. Most studies were descriptive in nature, with only three that used a case-control design. Majority of the studies were from urban areas, and addressed determinants rather than risk factors. Gender differences and age were predominantly reported, with more males committing suicide. Suicidal behavior was more common among individuals younger than 30 years of age. The three most common methods for suicides were hanging, poisoning and use of firearms. Mental illness as a risk factor for suicides was mentioned in only three studies.

**Conclusions:**

This review is the first attempt to synthesize available literature on suicidal behavior in Pakistan. The evidence is limited, and calls for more robust analytical research designs, along with a focus on risk factors.

## Background

In 2012, an estimated 804, 000 deaths by suicide occurred worldwide, representing an annual global age-standardized suicide rates of 11.4 per 100,000 population (15.0 for males and 8.0 for females) [1]. Suicide is considered the second leading cause of death in people between the ages of 15 and 29 years worldwide [2]. Although the numbers and rates differ significantly between countries, 75% of all global suicides occur in low and middle-income countries (LMICs) [1].

Research into suicidal behavior (which includes completed suicide, deliberate self-harm (DSH) and suicidal ideation) shows variations with respect to determinants, risk factors and motivations for such acts. Much of the research has been conducted in Western, industrialized countries where mental disorders appear to play a crucial role in suicidal behaviors, whereas in non- Western settings (particularly in South Asian cultures), interpersonal relationship problems appear to play a more critical role [3, 4].

Reported rates of suicide in several Asian countries appear to be higher than the average global rates with only two countries (India and China) contributing more than 45% of the global suicides [5]. Although there has been a percentage decrease in the rates of both countries, the numbers still remain high at 258,075 and 120,730 respectively [1, 5]. Pakistan is a low and middle income country with an estimated population of 200 million, making it the 6th most populous country in the world [6]. Ninety-seven percent of its population is Muslim and the Islamic religion plays an important role in peoples’ daily lives [7]. Approximately 50% of its population is under the age of 25 years [6]. The country has four provinces (Punjab, Sindh, Balochistan and Khyber Pakhtunkhawa), several languages, cultures, sub-cultures, ethnicities and religious sects. Since its independence, the country has faced major challenges of an unstable political system and poor governance and the country’s social and health indicators remain consistently poor [8]. Prevalence rates of common mental disorders (CMDs) put the figure as high as 34% [9].

Suicidal behavior remains an under-researched and under-studied subject in Pakistan [10]. Official mortality statistics on suicide are not available since they are not part of the national vital registration system nor reported to the WHO. Over the last couple of decades, there have been a growing number of studies on suicide and DSH that draw attention to the fact that suicidal behavior is being recognized as a serious public health problem [11, 12]. However, these are individual level studies that make it difficult to get a national picture of suicidal behavior. The recently published WHO report on suicide estimated that in 2012, there were 13,377 suicides (females 7085; males 6021) in Pakistan, with rates of 7.5 per 100,000 [1]. This is an increase of 2.6% in rates from the year 2000 [1]. WHO also estimates that for every suicide there are at least 10–20 acts of DSH. By this estimate, there may be between 130,000 to 270,000 acts of DSH in Pakistan annually [1].

Under-reporting and lack of research may occur due to criminalization of suicidal behavior in Pakistan. The Pakistan Penal Code (PPC) 325 states *“Whoever attempts to commit suicide and does any act towards the commission of such offence, shall be punished with simple imprisonment for a term which may extend to one year, (or with fine, or with both)”.* The law itself derives from the tenants of Islam, which strongly condemns suicidal behavior [13]. Under this law every case of suicide or DSH must be taken to one of the city/town’s government hospitals that is officially designated as a ‘medico-legal center’ (MLC). Only the MLCs are authorized to receive cases of suicide and DSH [13]. In reality however, people with DSH avoid going to the MLCs, for fear of legal complications and many seek treatment from private hospitals. Similarly, the latter, in order to protect the individual (and themselves) do not report DSH cases to the police, mislabeling them as either ‘accidental’ or give them a medical diagnosis. Also, as private medical care in Pakistan is quite expensive, many people leave against medical advice after emergency medical treatment [14]. Therefore, due to both financial reasons as well as legal, socio-cultural and religious stigma surrounding suicidal behavior in Pakistan, the underlying psychosocial issues remain largely unaddressed. The social consequences of suicidal behavior in Pakistan can be quite significant, with families often stigmatized and ostracized [14]. Stigmatization of suicidal behavior in Pakistani society may also be contributing to lack of research on the subject.

Considering the limited evidence available on suicidal behavior in Pakistan, we conducted a scoping review on the subject. The study aims to map the available literature on suicidal behavior in Pakistan, to provide a collective synthesis on the subject, allowing for future research and to inform policy for suicide prevention programs in the country. To the best of our knowledge, no previous study has employed this methodology for mapping suicidal behavior in Pakistan.

## Methods

We conducted a scoping review of suicidal behavior in Pakistan. Scoping review is recommended in settings where there is limited evidence on a subject, as it allows wider coverage of the topic [15].

The following operational definitions were utilized: i) Suicide is defined as an act of self-harm with a fatal outcome [16], ii) Deliberate self-harm (DSH) is defined as a non-fatal act of self-harm carried out with variable motivations [16], iii) Suicidal ideation is defined as thoughts, ideas and desire to commit suicide [17], iv) Determinants are a range of behavioral, biological, socio-economic factors influencing the health of the populations [18] v) Risk factors are characteristics or attributes within an individual that increases the likelihood of a disease [19].

For the review, we followed Arksey and O’Malley’s (2005) methodological framework, which includes the following six stages: (i) identification of the research question (ii) identification of relevant studies (iii) study selection (iv) data charting (v) data analysis and reporting the results (vi) consultation exercise [15].

The methods of this scoping review are described in light of the above mentioned six stages.

*Stage 1:* “What are the risk factors and determinants of suicidal behavior and methods employed in Pakistan?”

*Stage 2:* We developed a robust search strategy to identify relevant studies on the topic under review. We used a combination of key terms, including “Pakistan” and (“suicide” OR “attempted suicide” OR “parasuicide” OR “deliberate self-harm” OR “drug overdose” OR “self-poisoning” OR “acute poisoning” OR “organophosphate poisoning” OR “suicidal behavior”).

We searched ten different electronic databases, from the beginning of their timeframes, including Applied Social Sciences Index and Abstracts (ASSIA), Cochrane Trials Register (CRG), Cumulative Index to Nursing and Allied Health (CINAHL), National Library of Medicine Gateway (NLMG), ExcerptaMedica (EMBASE), National Library of Medicine’s MEDLINE (PUBMED), PSYCHINFO, Social Science Citation Index and Science Citation Index (SCI). Pakmedinet.com, a Pakistani medical publication website, was also searched for relevant literature. We also searched ‘grey literature’ which included unpublished theses and other reports. Databases were searched until December 2016.

The literature search was conducted by the head librarian (KM) who had access to the above databases via Aga Khan University and University of Alberta library resources.

### Eligibility criteria

The *inclusion criteria*:

- Any study design (primary research, case series or reports)
- Different types of suicidal behavior (including completed suicides, DSH and suicidal ideation)
- Suicidal behavior in both genders and across all ages
- Studies focusing on any of the three aspects: determinants, risk factors or methods employed

The *exclusion criteria*:

- Studies on Pakistanis residing outside the country (not included because of varied social context)

Variables of interest in each study included socio-demographic details, risk factors and methods employed in the suicidal act.

*Stage 3:* For study selection, screening was undertaken in two steps. Two reviewers (SS and DH) first screened the abstracts. After application of eligibility criteria, full text of the remaining articles were retrieved, which were also screened by the two reviewers.

*Stage 4:* The retained articles were entered in a data charting form, developed on Microsoft Excel, based on the literature review and research question. Key attributes of the data charting form included the site of the study, study design, number of subjects and outcome measures, along with specific information on risk factors, determinants and in cases of suicide and DSH, the methods employed. The form was pilot tested and modifications were made accordingly.

*Stage 5:* Information on distribution and nature of studies was extracted from data charting forms and reported after manual synthesis. The research question consisted of risk factors, determinants and methods employed that formed the basis for analysis. Main characteristics of studies on completed suicides and DSH were also reported in a tabular form. In cases where there was an overlap between risk factors and determinants, it was resolved via mutual consultation within the research team and reported separately.

*Stage 6:* We also undertook a consultation exercise with the subject expert (MMK) in order to add strength to our study. He also provided additional articles, which were included in the scoping review.

The subject expert also read the results and provided feedback, adding extra value to the review. Badger et al. (2000) recommend utilization of existing knowledge and networks generation of information on primary research [20].

## Results

**Fig. 1:**
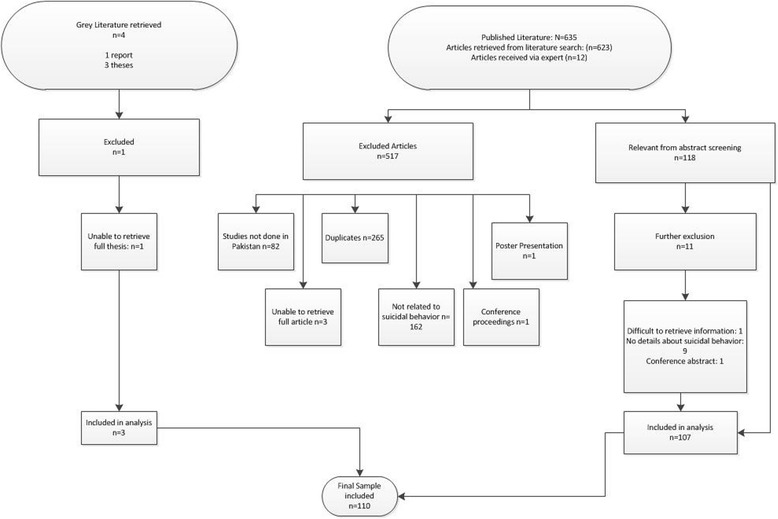
Consort flow chart

Through the ten search engines we initially retrieved 623 articles, of which 265 were duplicates and were excluded (Fig. 1). Two reviewers (SS and DS) reviewed the abstracts of the remaining 358 articles individually. In addition, 12 articles were also retrieved from the subject expert. Eighty-two studies not conducted in Pakistan or covering Pakistani population, one poster presentation, four conference proceedings, three articles that could not be retrieved (despite our best efforts) and 162 articles that did not cover suicidal behavior were excluded. One hundred and eighteen articles were read in full, out of which 11 were further excluded (nine were not relevant to our research question, one was a conference abstract and in one, information was difficult to extract), leaving us with 107 articles. To this number, one report and two PhD dissertations were added, giving a final total of 110 articles included for the final review. Table 1 summarizes the key characteristics of included original studies.

**Table 1:** Characteristics of original studies included in review

Author / Year	Study Design	Setting (City, Province)	Studies reporting on	Number of suicidal cases	Male to female ratio	Age of the study population	Method (three most common)	Psychiatric Diagnosis (% of sample)
Jamil et al. (1977) [56]	Retrospective case series (1976)	Karachi, Sindh	Unnatural deaths^a^	35 out of 53	N/A	N/A	Poisoning	N/A
Jamil et al. (1977) [40]	Retrospective case series (1976)	Karachi, Sindh	Unnatural deaths	96 out of 157	N/A	N/A	Poisoning	N/A
Ahmed et al. (1981) [57]	Retrospective case series (1974–1978)	Karachi, Sindh	Suicide & DSH	25 suicide 167 DSH	Suicide-1.27: 1 DSH- 5.8: 1	Suicide: 11–20 yrs.: 64% DSH: 21–20 yrs.: 50%	Suicide: drugs, poisons, sharp instruments DSH: drugs, poisons, sharp instruments	Suicide: 8% DSH: 1%
Noor et al. (1988) [58]	Retrospective case series (1984–1987)	Multan, Punjab	Unnatural deaths	20 out of 112	N/A	N/A	Poisoning	N/A
Jamil et al. (1990) [59]	Retrospective case series (1976–1985)	Karachi, Sindh	Unnatural deaths	1330 out of 1900	N/A	N/A	Poisoning	N/A
Javed et al. (1996) [24]	Cross-sectional (1992)	Lahore, Punjab	Suicidal ideation	27 out of 60	0.66: 1	N/A	N/A	N/A
Khan et al. (1996) [60]	Retrospective case series (1989–1992)	Karachi, Sindh	DSH	314	0.69:1	<30 yrs.: 71%	Poisoning, hanging, wrist slashing	31% affective disorder 6% schizophrenia
Khan et al. (1996) [25]	Retrospective case series (1989–1993)	Karachi, Sindh	DSH	382	0.69:1	<30 yrs.: 69%	Poisoning	N/A
Waseem et al. (1997) [61]	Prospective case series (1996)	Lahore, Punjab	Suicide	20	1.85: 1	N/A	Poisoning	N/A
Khan et al. (1998) [39]	Retrospective case series (1989–1994)	Karachi, Sindh	DSH	447	0.69:1	<30 yrs.: 62%	Poisoning	14%
Aziz et al. (1999) [62]	Retrospective case series (1993 & 1995)	Lahore, Punjab	Suicide	96	1.43: 1	20–30 yrs.: 49%	Hanging, firearms, burning	15%
Malik et al. (1999) [26]	Retrospective case series (1984–1996)	Lahore, Punjab	Unnatural deaths	2 out of 837	1:1	N/A	Cutting	N/A
Bashir et al. (2000) [63]	Retrospective case series (1994–1999)	Lahore, Punjab	Unnatural deaths	45 out of 91	N/A	N/A	Hanging	N/A
Rana et al. (2000) [64]	Retrospective case series (1984–1988)	Lahore, Punjab	Unnatural deaths	21	N/A	N/A	Poisoning	N/A
Khalid et al. (2000) [65]	Retrospective case series (1997–2001)	Karachi, Sindh	Suicide	1230	2.19: 1	21–30 yrs.: 47.21%	Gunshot, hanging, poisoning	9% males 5% females
Khan et al. (2000) [66]	Retrospective case series (1996–1997)	Karachi, Sindh	Suicide	306	2.1: 1	<30 yrs.: 82%	Poisoning, hanging, firearms	3%
Bunggush et al. (2000) [67]	Retrospective case series	Pakistan	Unnatural deaths	243 out of 408	N/A	N/A	Poisoning	N/A
Haider et al. (2001) [30]	Cross-sectional (2000)	Lahore, Punjab	DSH	100	0.67:1	15–24 yrs.: 36%	Poisoning	34%
Agha et al. (2001) [68]	Case-control (1999)	Karachi, Sindh	DSH	Case: 72 Control: 72	Case: 1:1 Control: 1:1	Mean age in both: 20–29 yrs	Benzodiazepines	100% in case group
Khan et al. (2001) [69]	Case-control (1998)	Peshawar, KPK	Suicidal ideation	4 out of 50	N/A	N/A	N/A	N/A
Ghazanfar et al. (2001) [70]	Retrospective case series (2006–2008)	Rawalpindi, Punjab	Unnatural deaths	27 out of 91	1.7: 1	N/A	Organophosphorus poisoning	N/A
Haider et al. (2002) [31]	Cross-sectional (2000)	Lahore, Punjab	DSH	385	0.81: 1	15–35 yrs.: 66%	Poisoning, cutting, firearms	52%
Ahmad et al. (2002) [71]	Cross-sectional (1996–2000)	Multan, Punjab	Unnatural deaths	193 out of 370	N/A	N/A	Poisoning	N/A
Haider et al. (2002) [50]	Cross-sectional (2002)	Lahore, Punjab	DSH	147	0.88: 1	Mean age (males): 30.5 yrs. Mean age (females): 29.4 yrs	Poisoning	71%
Sultana et al. (2002) [72]	Retrospective case series	Karachi, Sindh	Unnatural deaths	51 out of 632	N/A	N/A	Hanging, drowning, cutting	N/A
Hasan et al. (2002) [73]	Retrospective case series (1998–2001)	Rawalpindi, Punjab	Unnatural deaths	101 out of 181	N/A	N/A	N/A	N/A
Saeed et al. (2002) [74]	Retrospective case series (1998–2001)	Faisalabad, Punjab	Suicide	95	2.44: 1	20–29 yrs.: 43.1%	Hanging, Firearms, poisoning	N/A
Khan et al. (2003) [75]	Retrospective case series (2001)	Quetta, Baluchistan	DSH	46	100% females	16–25 yrs.: 65.2%	Poisoning	78%
Ali et al. (2003) [76]	Retrospective case series (2001)	Peshawar, KPK	Unnatural deaths	2 out of 679	100% females	N/A	Firearms, sharp weapons,	N/A
Ali et al. (2003) [77]	Retrospective case series (1997–2001)	Peshawar, KPK	Unnatural deaths	9 out of 52	N/A	N/A	Poisoning	N/A
Ahmed et al. (2003) [21]	Retrospective case series (1995–2001)	Karachi, Sindh	Suicide	1379	1.7: 1	21–30 yrs.: highest	Poisoning, hanging, firearms	N/A
Bashir (2003) [22]	Retrospective case series (1991–2000)	Peshawar, KPK	Suicide	39	2.9: 1	20–29 yrs.: highest	Firearms, hanging	N/A
Safdar et al. (2003) [78]	Retrospective case series	Sukkur, Sindh	Unnatural deaths	14 out of 26	N/A	N/A	Poisoning	N/A
Farooqi (2004) [79]	Cross-sectional (2003)	Lahore, Punjab	Suicidal ideation	50 out of 100	1.5: 1	N/A	N/A	40% depression 24% schizophrenia
Valika et al. (2004) [80]	Cross-sectional (2003)	Pakistan	Suicidal behavior	19	1: 0.88	N/A	Poisoning	N/A
Farooqi et al. (2004) [81]	Cross-sectional (2003)	Karachi, Sindh	DSH	50	1.5: 1	11–20 yrs.: 40%	Poisoning	N/A
Waseem et al. (2004) [82]	Prospective case series (2003–2004)	Lahore, Punjab	Unnatural deaths	31 out of 70	N/A	N/A	Poisoning	N/A
Shoaib et al. (2005) [34]	Prospective case series (2004)	Lahore, Punjab	Suicide & DSH	107	1.22:1	21–30 yrs.: 49%	Poisoning	9%
Khoker et al. (2005) [83]	Cross-sectional (2004)	Karachi, Sindh	Suicidal ideation	68 out of 217	0.87:1	N/A	N/A	N/A
Hussain et al. (2005) [37]	Retrospective case series (1996–2002)	Karachi, Sindh	Unnatural deaths	40 out of 50	N/A	N/A	Poisoning	N/A
Asif et al. (2005) [32]	Cross-sectional (2004)	Lahore, Punjab	DSH	1390	2.22: 1	21–30 yrs.: 36%	Poisoning	N/A
Rasheed et al. (2005) [29]	Prospective case series (2004)	Rawalpindi, Punjab	Suicidal ideation	Under-trial prisoners: significant relationship with suicidal behavior	100% males	N/A	N/A	N/A
Kermani et al. (2006) [84]	Retrospective case series (2004)	Karachi, Sindh	DSH	150	0.75: 1	21–25 yrs.: 41%	Poisoning	N/A
Aziz (2006) et al. [85]	Cross-sectional (2002–2004)	Larkana, Sindh	Suicide	52	2.03: 1	30–39 yrs.: 44.2%	Firearms, hanging, burning	73%
Khan (2006) [10]	Retrospective case series (1985–1999)	Karachi, Sindh	Suicide	2568	2.78: 1	N/A	Poisoning, hanging & drowning	N/A
Suliman et al. (2006) [86]	Prospective case series (2002–2003)	Bahawalpur, Punjab	Unnatural deaths	111 out of 143	N/A	N/A	Poisoning	N/A
Tahir et al. (2006) [54]	Prospective case series (2003–2004)	Sukkar, Sindh	Unnatural deaths	17 out of 24	N/A	N/A	Poisoning	N/A
Rathore et al. (2007) [87]	Prospective case series (2006–2007)	Lahore, Punjab	Suicide & DSH	50	1.27: 1	21–30 yrs.: 44%	Poisoning	N/A
Ahmad et al. (2007) [88]	Prospective case series (2002–2003)	Islamabad, Punjab	Unnatural deaths	9 out of 142	N/A	N/A	Burns	N/A
Shaikh et al. (2008) [23]	Retrospective case series	Hyderabad, Sindh	Unnatural deaths	99 out of 111	N/A	N/A	Poisoning	N/A
Raja et al. (2008) [48]	Cross-sectional (2007)	Karachi, Sindh	Unnatural deaths	9539 out of 27,254	N/A	N/A	Poisoning	N/A
Babar et al. (2008) [35]	Retrospective case series (2002)	Islamabad, Punjab	Suicide	227	2.3: 1	25–45 yrs.: 63%	Poisoning, shooting	3%
Syed et al. (2008) [49]	Retrospective case series (1990–2006)	Karachi, Sindh	DSH	69	0.59: 1	> 14 yrs.: 89.9%	Poisoning, hanging, firearms	5.8%
Shahid et al. (2008) [89]	Retrospective case series (2004)	Karachi, Sindh	DSH	98	0.58: 1	Mean age: 23.5 yrs	Drug ingestion, organophosphate poisoning	N/A
Karamaliani et al. (2008) [90]	Cohort study	Hyderabad, Sindh	DSH	2 out of 2324	N/A	N/A	N/A	N/A
Patel et al. (2008) [91]	Retrospective case series (2002–2006)	Karachi, Sindh	DSH	202	0.69:1	Mean age: 17 yrs	Benzodiazepines, anti-depressants	58.4% depression, 10.9% bipolar disorder
Khan et al. (2008) [28]	Case-control (2003)	Karachi, Sindh	Suicide	100 cases 100 controls	4.88: 1	N/A	Hanging, poisoning & firearms	96%
Khurram et al. (2008) [33]	Cross-sectional (2006)	Rawalpindi, Punjab	DSH	60	0.72: 1	N/A	Poisoning	% not mentioned
Shaikh et al. (2008)	Retrospective case series (2004–2006)	Karachi, Sindh	Unnatural deaths	99 out of 111	N/A	N/A	Poisoning	N/A
Zakiullah et al. (2008) [92]	Retrospective case series (1997–2002)	Karachi, Sindh	DSH	283	0.66: 1	12–15 yrs.: 22.2%	Poisoning, cutting, burning	50.3%
Khan (n.d) [93]	Cross-sectional	Chitral, KPK	Completed Suicides	32	100% females	16–20 yrs.: 59%	N/A	0%
Farooq et al. (2009) [94]	Retrospective case series (1999–2008)	Rawalpindi, Punjab	Unnatural deaths: 5.06% suicidal	9	N/A	N/A	Burns	N/A
Ayub (2009) [95]	Cross-sectional	Pakistan	Suicidal ideation	N/A	N/A	N/A	N/A	Hopelessness linked to suicidal ideation
Khan et al. (2009) [27]	Cross-sectional (2000–2004)	Ghizer, KPK	Suicide	49	100% females	18–25 yrs.: 59%	Drowning, Poison, hanging	N/A
Shahid et al. (2009)	Retrospective case series (2008)	Karachi, Sindh	DSH	98	0.59: 1	Mean age: 23.5 yrs	Benzodiazepines, organophosphate poisoning, alcohol poisoning	24.2%
Asad et al. (2010) [117]	Cross-sectional	Hyderabad, Sindh	Suicidal ideation & attempts	41 attempted suicide 150 suicidal thoughts	100% females	N/A	N/A	18%
Farooq et al., (2010) [42]	Retrospective case series (2007–2008)	Rawalpindi, Punjab	DSH	Police reports: 7 ED: 33	Police reports: 13.2: 1 ED: 5.6:1	ED: 15–56 yrs.: 77.3%	Poisoning	N/A
Rizwan (2010) [97]	Case-control	Karachi, Sindh	Suicidal ideation	120 in case, 120 in control	Case: 2.15: 1 Control: 1:1	Mean age in case: 22 yrs. Mean age in control: 21 yrs	N/A	100% in case group
Tahir et al. (2010) [55]	Retrospective case series (2001–2008)	Jamshoro, Sindh	DSH	154	0.55: 1	Mean age: 31.21 yrs	Burning	67%
Faruqui et al. (2011) [98]	Cross-sectional (2009)	Islamabad, Punjab	Suicidal ideation	15 out of 50	N/A	N/A	N/A	N/A
Naz (2012) [99]	Retrospective case series (2010)	Karachi, Sindh	Suicide	10	N/A	N/A	N/A	N/A
Ali et al. (2012) [100]	Cross-sectional	Karachi, Sindh	Suicidal Ideation	28	100% females	Not mentioned	N/A	% not mentioned
Kumar et al. (2012) [101]	Retrospective case series (2010–2012)	Larkana, Sindh	Unnatural deaths	91 out of 10,130	N/A	N/A	N/A	N/A
Lakhair et al. (2012) [102]	Prospective case series	Hyderabad, Sindh	Unnatural deaths	58 out of 70	N/A	N/A	N/A	N/A
Mirza et al. (2012) [103]	Retrospective case series (2005–2010)	Karachi, Sindh	Unnatural deaths	7 out of 61	N/A	N/A	N/A	N/A
Ali (2012) et al. [104]	Prospective case series (2008)	Karachi, Sindh	Unnatural deaths	65 out of 100	N/A	N/A	Organophosphorous poisoning	N/A
Kehtran et al. (2012) [105]	Retrospective case series (1998–2000)	Barkhan, Baluchistan	Unnatural deaths	2 out of 268	N/A	N/A	Firearms	N/A
Riaz et al. (2012) [106]	Cross-sectional (2012)	Karachi, Sindh	DSH	9	N/A	N/A	Cutting	
Tahir et al. (2013) [107]	Cross-sectional (2011)	Mianwalli, Punjab	DSH	108	3.54: 1	21–30 yrs.: 50.5%	Poisoning & firearms	33%
Khalil et al. (2013) [96]	Retrospective case series (2009–2012)	Peshawar, KPK	Unnatural deaths	66 out of 2365	12.2: 1	20–40 yrs	Firearm, blunt trauma	N/A
Ali (2013) et al. [108]	Cross-sectional	Karachi, Sindh	Suicidal ideation	759: 58.8% had suicidal thoughts	100% females	N/A	N/A	% not mentioned
Ayub et al. (2013) [109]	Cross-sectional (2012)	Lahore, Punjab	Suicidal ideation	636 out of 650	100% females	N/A	N/A	93%
Raza et al. (2014) [110]	Retrospective case series (2009–2013)	Lahore, Punjab	Unnatural deaths	3 out of 31	N/A	N/A	Knife, razor blade	N/A
Shaikh et al. (2014) [111]	Prospective case series	Karachi, Sindh	Suicidal ideation	171	N/A	N/A	N/A	Anxiety statistically significant with suicidal ideation
Salman et al. (2014) [112]	Prospective case series (2012)	Peshawar, KPK	DSH	45	0.8: 1	N/A	N/A	N/A
Saiq et al. (2014) [113]	Prospective case series (2010–2012)	Islamabad, Punjab	DSH	93	0.24: 1	<35 yrs.: 96%	Burning	1.07%
Osama et al. (2014) [114]	Cross-sectional (2013)	Karachi, Sindh	Suicidal ideation	118 out of 331	0.70: 1	Mean age: 20.73 yrs	N/A	N/A
Husain et al. (2014) [36]	Randomized control trial (2010–2012)	Karachi, Sindh	DSH	221	0.45: 1	Mean age: 23.1 yrs	Poisoning	N/A
Shagufta et al. (2015) [115]	Prospective case series (2014)	Peshawar, KPK	Suicidal ideation	91 out of 415	100% males	11–18 yrs.: 100%	N/A	0.24%
Rao et al. (2015) [116]	Cross-sectional (2009)	Pakistan	Suicidal ideation	334 out of 4583	N/A	N/A	N/A	N/A
Shahid et al. (2015) [46]	Case-control study (2011–2012)	Karachi, Sindh	DSH	201 cases 201 controls	0.7: 1	N/A	N/A	9% depression

^a^Unnatural deaths (homicidal, accidental & suicidal)

Forty-eight studies were conducted in Sindh (39 from Karachi), 31 in Punjab (16 from Lahore and 11 from Rawalpindi/Islamabad), 9 from KPK (7 from Peshawar) and 2 in Baluchistan. The remaining 20 were describing trends from all over Pakistan.

The reviewed articles consisted of 25 cross-sectional surveys, fourteen prospective case-series and 45 retrospective case series. There were only five case-control studies and only one randomized control trial. 19 articles were literature reviews, commentaries or editorials. One paper was a population-based cohort study.

Rates of suicide or DSH were mentioned in only six studies. Two studies determined rates through descriptive study designs, whereas two from literature review and archival researches. Two studies determined rates of suicides for individual cities [21, 22]. Suicide rates varied from 0.43**/**100,000/year (for the years 1991–2000) in Peshawar to 2.86**/**100,000 (in 2006) in Rawalpindi. Gender-specific rates showed lowest and highest rates for men were 0.61/100,000 in Peshawar and 5.2**/**100,000 in Rawalpindi, respectively and for women 0.23**/**100,000 in Peshawar and 1.77**/**100,000 in Larkana, respectively [10, 23]. Average suicide rates for the years 1985–1999 in the province of Sindh was calculated to be 1.15/100,000 population v[10].

### Determinants

Gender differences were reported in almost all studies of both completed suicides and DSH. Collectively, more males than females committed suicide. However, 18 studies on DSH reported more females than males, while the trend was reversed in 10 studies. Suicidal ideation was more common in females than males. Data on gender and marital status was not disaggregated in all studies but in studies where disaggregation was done, they showed there are more married than single females in both suicides and DSH [24–26].

A number of studies focused on suicidal behavior in Pakistani women exclusively: six studies on suicidal ideation, three on completed suicides and two on DSH. A study on suicide in women in the Ghizer district in the remote Northern areas of Pakistan showed average annual rates of 14.89/100,000 for the years 2000–2004, with rates in those of over 15 years of age as 33.22/100,000. Important correlates were domestic violence and high levels of psychiatric morbidity [27].

More than half of the studies reported the age of their study sample but used different age ranges. Both suicide and DSH appears to be more common among the young, with majority being less than 30 years of age.

‘Unemployment’ or ‘financial hardship’ as a cause of suicidal behavior was also mentioned in a few studies (8 studies on suicide, 8 on DSH and one on suicidal ideation). Unemployment rates varied from 4% to 39% in suicides and from 4% to 86% for DSH. A case-control study of 100 suicides and matched living controls in Karachi showed that 39% of cases were unemployed as compared to 17% of controls [28].

Two studies (one for DSH and one for suicidal ideation) reported on the association between level of income and suicidal behavior. 46%–67% of those who engaged in suicidal behavior had an income of less than Rs. 6000 per month (US $60) [23, 29]. In four DSH studies, 67% to 91% belonged to lower socio-economic class, whereas only 1% to 4% belonged to upper class [30–33].

Level of education was reported in 14 studies: 3 for suicidal ideation, 2 for completed suicides and 9 for DSH and showed that suicidal behavior was more common among those who had little or no education, with rates varying from 30% to 60%. In the case-control study on suicide, 21% of cases were uneducated as compared to 4% of controls [28].

Other determinants that were reported in some studies included occupation. Housewives were 20% to 60% while students were 4% to 17.5% of the study sample [28, 34, 35].

### Risk factors

While determinants for suicidal behavior were mentioned in several studies (above), only two studies (both were case-control design) investigated risk factors for suicide and DSH specifically. In the DSH study, ‘mental illnesses, low socio-economic status and loneliness’ were found to the risk factors for patients presenting to three emergency departments of Karachi, while the case-control psychological autopsy study of suicide identified six factors: life-events, disrupted social network, level of academic qualification, marital status, ethnicity and depression, that were linked to very high Population Attributable Risk Fractions (PARFs) ranging from 37% for education to 97% for life events [28].

There has been only one intervention trial for suicidal behavior in Pakistan, that compared the efficacy of a brief psychological intervention (delivered following an episode of self-harm) with treatment as usual (TAU) [36]. Patients in the intervention group showed statistically significant improvement on the Beck Scale for Suicide Ideation and Beck Hopelessness Inventory, which was sustained at 3 months [36].

### Methods for suicidal behavior

Methods of suicide and DSH were reported in more than 50% of the studies. The three most common methods for suicide in Pakistan appear to be hanging, ingesting poisons and use of firearms [21, 36–38]. Amongst ‘poisons’, the majority of victims used insecticides and pesticides, (that contain organophosphate (OP) compounds), that are available in most homes in the urban areas and used in agriculture in the rural areas of Pakistan respectively. Despite being freely available over-the-counter, medications (including analgesics and psychotropics) are not used widely as means to commit suicide. In contrast, self-poisoning with medications (particularly benzodiazepines) was the most common method in DSH in the urban areas [39]. On the other hand, self-poisoning was more common in both suicide and DSH in semi-urban or rural areas [40].

## Discussion

This scoping review is the first attempt to synthesize the available literature on suicidal behavior in Pakistan. The results reveal that there are significant gaps in evidence.

Majority of the studies are from the main urban centers of the country, and there is dearth of data from rural areas, despite the fact that almost two-thirds of the population in Pakistan lives in rural areas.

Most of the studies reviewed were descriptive in nature (majority were case series and cross-sectional surveys that do not allow for the establishment of relationships), with only five case-control studies.

A number of studies were on “unnatural deaths”, in which the *manner of death* (for example, whether accidental, homicidal or suicidal) was not disaggregated, making it difficult to study the characteristics (such as age, gender or marital status) of the suicidal group separately.

Lack of official statistics for suicide prevents the problem being recognized. Since suicide and DSH remain criminal acts in Pakistan, this further poses a challenge in accurate data collection [41]. Studies have reported a discrepancy in reported rates between newspaper reports and police data [42]. In Pakistan, given the strong religious views against suicide, decriminalization may be perceived as endorsing suicidal behavior as a way out of one’s problems [43]. However, as the WHO estimates show (an increase of 2.6% in suicide rates between 2000 and 2012) religion may not be as strong a deterrent as previously perceived [1]. Decriminalization would have the effect of partly decreasing the stigma surrounding the act, thus allowing people to seek help without fear of prosecution or harassment by the law enforcement authorities. Although the Mental Health Act of 2001 has made some provisions for the protection of those who attempt suicide by including that *“a person who attempts suicide shall be assessed by an approved psychiatrist and if found to be suffering from a mental disorder shall be treated appropriately under the provisions of this Ordinance”*, this has yet to translate into practice. More recently, there is some indication that suicidal behavior may be decriminalized in Pakistan [44]. Indeed if this was to happen, it would be a major step in eliminating the stigma and addressing the psychological needs of suicidal persons in the country.

Despite the poor quality of many studies one consistent finding that emerges in our review is that gender is an important determinant for suicide and DSH in Pakistan, especially when considered in the context of marital status. There was a larger representation of married women compared to single women or married or single men. It appears that unlike the West, where it is protective, marriage is a risk factor for psychiatric morbidity and suicidal behavior for Pakistani women [45]. Associated factors include early age of marriage, lack of autonomy in choice of male partner (‘arranged marriage’), pressure to have children early in the marriage, desire for a male offspring, curtailment of education, economic dependence on husband, joint or extended family system and domestic violence [46]. These factors put many young married women in Pakistan in a highly disadvantaged position and many resort to suicidal behavior as a way to express their distress. Other studies report that being a female in Pakistan is in itself a risk factor for suicide and DSH, with lack of employment being a significant determinant, and its association with issues of control and empowerment [47].

This scoping review shows that majority of individuals who engaged in suicidal behavior are below the age of 30 years, underscoring the need to address mental health and other issues faced by young people in Pakistan. Conversely, suicide and DSH was uncommon among the elderly, a finding that is in sharp contrast to studies from the West [48]. Part of the explanation may lie in the fact that in Pakistan, few elderly people are socially isolated or live on their own where they have to fend for themselves. The majority are looked after by their families, who provide both physical as well as financial support.

Unemployment and financial hardships appear to be strongly correlated with both suicide and DSH, particularly among males in Pakistan [49]. Unemployment, poor economic conditions and rising poverty are macro level factors in suicidal behaviors that need to be addressed at policy levels [50].

Low educational attainment strongly correlated with both suicide and DSH in the studies we reviewed. This is likely mediated through poor stress coping abilities, inability to compete for jobs or acquire greater social standing [51].

Our scoping review showed that poisoning is the second most common method (after hanging) in suicides in Pakistan. Amongst poisons, organophosphorous compounds feature highly in both DSH and suicide. These substances are highly toxic due to their anticholinesterase effects, leading to a high case-fatality index (even in cases with low suicidal intent). The free availability of pesticides in rural areas pose particular risk to those working in the agriculture sector in Pakistan, shown by their increased use in cases of self-poisoning. This is compounded by the absence of quality medical care in cases of poisonings in Pakistan.

As part of its suicide prevention strategy, the WHO lists restricting access to toxic agents as one of its recommendations. Trials of ‘locked boxes’ (that restrict access to toxic pesticides in crisis situations) undertaken in India and Sri Lanka have given encouraging results [52]. WHO also recommends use of less toxic pesticides for agricultural purposes, i.e. Class I OP pesticides and Class II endosulfan [52]. Both these measures could be applied for suicide prevention in Pakistan.

The increased use of firearms in suicides in Pakistan reflects their growing availability in the country, estimated at more than 20 million (of which only 7 million are registered), with almost 13,000 annual homicides [53]. There is urgent need for firearm control in the country.

This paper highlights the lack of studies on risk factors for suicide and DSH in Pakistan. Mental illness as a risk factor is addressed in a small minority of studies reviewed. This may be due to the fact that most studies in our review were conducted by non-mental health professionals [33, 54, 55], who are not sensitized to study mental illness in suicidal behaviors. Data is also biased away from mental illness as a risk factor due to stigma surrounding mental illness and suicidal behavior in itself.

There are serious lacunae and weaknesses in the current system of registration and diagnosing of suicidal behavior in Pakistan. There is lack of standardized system of certifying suicidal deaths across the country. Processes to investigate suicides are weak and influenced by socio-political and cultural factors. For example due to stigma it is not uncommon for families to have the suicidal death registered as an accident or a medical condition [2]. Conversely, many cases of homicide (particularly where the woman is set on fire by the husband and/or in-laws) are labeled as self- immolation suicides. Therefore, there is underreporting of suicidal behaviors in the country, the true extent of which is difficult to determine.

Currently, data on suicide and DSH is neither included in the National Health Morbidity Statistics nor reported to the WHO. As a consequence national rates of suicide are not known [1]. On the other hand, as several health-facility based studies in our review show, cases of DSH do report to health facilities across the country. With decriminalization and a proper system for recording and collating data, it is possible to get a better picture of the problem in the country.

### Recommendations

There is an urgent need for DSH and suicide mortality statistics to be collected through a standard system of registration, recording and diagnosis, at all town/city, district and provincial levels throughout the country. The information obtained can be used for epidemiological-analytical, intra-country and cross-national studies. A mandatory reporting of suicide mortality statistics to the WHO would help improve data collection and surveillance of suicides and DSH in Pakistan.

Based on this review’s findings, there emerges a need for improving the system of investigating and diagnosing suicides in the country. Training and education of key personnel involved in the process is vital, including the police, medico-legal officers, forensic medical specialists as well as general/family physicians.

Prohibiting use of the more toxic pesticides and replacing them with less toxic ones can help prevent many suicidal deaths, especially those with a low suicidal intent. There is need for improved emergency medical treatment for DSH victims, particularly those of self-poisoning with toxic agents.

This scoping review signifies that the existing evidence on suicidal behavior is limited in the context of Pakistan. Therefore, more robust analytical research designs such as case control and psychological autopsy methods are needed with a focus on risk factors, particularly mental illness. There is also need for intervention studies for prevention of suicidal behaviors in Pakistan.

All of the above recommendations demonstrate the need to have suicide prevention programs with an integrated research agenda in the existing health systems of Pakistan.
